# The mediating role of quality of life in the relationship between teachers’ participation in physical activity and empathic and social self-efficacy

**DOI:** 10.3389/fpsyg.2026.1781543

**Published:** 2026-03-11

**Authors:** Muhammed Ozkan Turhan, Ramazan Arslanboga, Sultan Yavuz Eroglu, Mustafa Can Koc, Lara Carneiro, Laurentiu-Gabriel Talaghir, Gabriel Marian Manolache, Teodora Mihaela Iconomescu, Florentina Cristea, Anamaria Berdila

**Affiliations:** 1 Faculty of Sports Sciences, Mus Alparslan University, Mus, Türkiye; 2 Faculty of Sports Sciences, Bingöl University, Bingöl, Türkiye; 3 Faculty of Sports Sciences, Istanbul Gelisim University, Istanbul, Türkiye; 4 Physical Education Department, College of Education, United Arab Emirates University, Abu Dhabi, United Arab Emirates; 5 Faculty of Physical Education and Sport, Dunarea de Jos University of Galati, Galati, Romania

**Keywords:** empathic, physical activity, self-efficacy, social, teacher

## Abstract

**Background:**

There is no study in the literature examining whether quality of life plays a mediating role in the relationship between teachers’ participation in physical activity and self-efficacy.

**Objective:**

The aim of this study is to examine the mediating role of quality of life in the relationship between teachers’ participation in physical activity and their perceived empathic and social self-efficacy.

**Methods:**

The participants were between 23 and 40 years old, with a mean age of 29.3 ± 7.79 years. As data collection instruments, the International Physical Activity Questionnaire-Short Form adapted into Turkish by Öztürk (2005), the Quality of Life of Employees Scale developed by Stamm (2005) and adapted into Turkish by Yeşil, Ergün, Amasyalı, Er, Olgun, and Aker (2010), and the Perceived Empathic and Social Self-Efficacy Scale developed by Di Giunta et al. (2010) and adapted into Turkish by Akın and Başören (2015) were used. Construct validity of the scales was tested using confirmatory factor analysis, and the analyses were conducted via SPSS, AMOS, and the PROCESS Macro plugin.

**Results:**

The results revealed moderate positive relationships between job satisfaction and both empathic and social self-efficacy.

**Conclusion:**

Mediation findings indicated that quality of life (job satisfaction) played a significant mediating role in the relationship between physical activity and empathic self-efficacy, as well as in the relationship between physical activity and social self-efficacy.

## 1 Introduction

In modern educational settings, teachers’ psychological resources affect not only their individual well-being but also students’ learning processes and the overall success of educational institutions. Therefore, the interaction between teachers’ self-efficacy perceptions, quality of life, and physical activity levels has become increasingly important (Wang et al., 2024). This study examines two-dimensional self-efficacy perception models, namely empathic and social self-efficacy, and observes that quality of life functions as a mediating variable in the relationship between physical activity and self-efficacy through the subscale of job satisfaction.

Self-efficacy theory refers to an individual’s belief in their ability to succeed in a task (Bandura, 1997; cited in Schwarzer, 2014). In educational contexts, this belief can play a decisive role in teachers’ classroom management, communication with students, and professional decision-making. Empathic self-efficacy, in particular, influences teachers’ capacity to perceive students’ emotional states and to develop appropriate pedagogical approaches (Di Fabio and Kenny, 2016). Social self-efficacy, on the other hand, supports individuals’ confidence in social interactions and their effective participation in these interactions (Zhang et al., 2025). These two dimensions of self-efficacy should be considered in relation to quality of life and physical activity when integrating teachers’ psychological resources.

Quality of life refers to an individual’s overall satisfaction with life, based on the degree to which their physical, psychological, and social needs are met. In the educational context, professional satisfaction, an aspect of this concept, includes elements such as teachers’ satisfaction with their profession, sense of justice, and job satisfaction (İlke, 2025; Judge et al., 2001). Teachers with high levels of job satisfaction are in a more advantageous position in terms of emotional energy and commitment (Van Horn et al., 2004). Furthermore, the literature shows that job satisfaction is a psychological resource that can establish a positive relationship between teachers’ job satisfaction and their perceptions of self-efficacy (Skaalvik and Skaalvik, 2017). According to this perspective, the effect of physical activity may be reflected in self-efficacy perceptions through professional satisfaction.

Physical activity not only protects physical health but is also an important factor that enhances psychological well-being, stress-coping skills, and life satisfaction (Biddle and Asare, 2011). There are also studies in the literature examining the relationship between physical activity and various psychological variables (Koç, 2025a, 2025b; Elmas et al., 2021; Demirer and Erol, 2020; Han, 2025). Research in education demonstrates that teachers’ participation in regular physical activity increases their job satisfaction and professional fulfillment levels (Maher et al., 2014). These findings point to the potential of physical activity to enhance professional fulfillment and suggest that it may positively influence perceptions of self-efficacy.

The lifelong maintenance of physical activity is of critical importance for enhancing overall health and quality of life (Koç, 2025a,b). Physical activity can support individuals’ self-confidence, resilience, and social interaction skills. According to self-efficacy theory, individuals learn from previous successful experiences and can strengthen their self-efficacy perceptions by reinforcing these experiences through regular physical activity (Bandura, 1997; cited in Schwarzer, 2014). For example, Aktaş (2018) found a significant relationship between the empathic skills and social problem-solving abilities of physical education and sports teacher candidates. This finding suggests that physical activity levels may have positive effects on empathic skills. Furthermore, Chen et al. (2025) revealed that the relationship between social support and social skills can influence individuals’ perceptions of social self-efficacy. In this context, social interactions and supportive relationships demonstrate the potential to strengthen individuals’ social skills and perceptions of self-efficacy.

Job satisfaction appears to be a direct source of self-efficacy perception. Teachers with a high quality of life attribute meaning to their duties, maintain a balance in their emotional resources, and may have a stronger sense of self-efficacy in social interactions (Lampropoulou, 2024). Similarly, a positive relationship has been observed between teachers’ self-efficacy perceptions and job satisfaction; this relationship has been shown to strengthen teachers’ social self-efficacy perceptions by increasing their confidence levels in social interactions through satisfaction with their jobs (Xiao and Zheng, 2025). These findings emphasize the impact of job satisfaction on social skills and confidence. Therefore, professional satisfaction should be evaluated not only for its direct effects on empathic and social self-efficacy but also as a mediating variable.

Based on this literature, our hypotheses are proposed in the text with their theoretical justifications as follows:

*H_1_*: Physical activity level has a significant and positive effect on quality of life (job satisfaction). This hypothesis is based on research indicating that physical activity is a factor that enhances not only physical health but also psychological resilience and job satisfaction. Regular physical activity increases individuals’ energy levels and overall life satisfaction by improving their ability to cope with stress. Dallmeyer et al. (2023) demonstrated that weekly leisure-time physical activity significantly increased individuals’ job satisfaction and directly contributed to their quality of life. This finding supports the assumption that physical activity may be an important determinant of job satisfaction.

*H_2_*: Quality of life (job satisfaction) has a significant and positive effect on empathic self-efficacy. This hypothesis is based on the assumption that job satisfaction can enhance individuals’ emotional balance and self-awareness, thereby developing their empathic skills. Individuals who are satisfied with their professional life nurture positive feelings toward their work and can more easily recognize the emotional needs of those around them. Skaalvik and Skaalvik (2017) stated that teachers’ levels of job satisfaction are positively related to their ability to understand students’ emotional states and to approach them empathically. In this regard, it can be said that as quality of life increases, empathic self-efficacy also strengthens.

*H_3_*: Physical activity level has a significant and positive effect on empathic self-efficacy. This hypothesis is based on the assumption that regular physical activity supports emotional awareness and empathy skills. Physical activity can strengthen individuals’ capacity to cope with stress, maintain emotional balance, and increase sensitivity in social interactions. Kuzmić et al. (2025) found that physically active teachers have higher levels of empathic self-efficacy in understanding their students’ emotions and developing appropriate responses to them. In this regard, it can be said that empathic self-efficacy develops as the level of physical activity increases.

*H_4_*: Quality of life (job satisfaction) mediates the relationship between physical activity and empathic self-efficacy. This hypothesis is based on the assumption that physical activity indirectly strengthens teachers’ empathic self-efficacy by improving their quality of life. Regular physical activity increases individuals’ job satisfaction, which in turn contributes to emotional balance, self-awareness, and heightened empathic sensitivity. Deng et al. (2023), in their study examining the relationship between physical activity, life satisfaction, and psychological well-being, revealed that life satisfaction plays a mediating role in this process. In this regard, it can be said that quality of life is an important mediating variable on the path to empathic self-efficacy.

*H_5_*: Physical activity level has a significant and positive effect on quality of life (job satisfaction). This hypothesis is supported by findings indicating that regular physical activity increases job satisfaction and overall life satisfaction. Physical activity boosts individuals’ energy levels, strengthens their ability to cope with stress, and contributes to maintaining emotional balance in their work life. Parallel to H_1_, this model also predicts that physical activity will positively affect teachers’ quality of life.

*H_6_*: Quality of life (job satisfaction) has a significant and positive effect on social self-efficacy. Individuals with high levels of satisfaction may feel more competent and effective in social settings and may take on a more active role in social interactions. Within the framework of social cognitive theory, it is emphasized that there is a reciprocal reinforcement relationship between life satisfaction and self-efficacy perception. In this regard, it can be said that as quality of life increases, individuals’ levels of social self-efficacy also rise (Wang et al., 2024).

*H_7_*: Physical activity level has a significant and positive effect on social self-efficacy. Physical activity not only improves physical health but also develops individuals’ social participation and communication skills. Active individuals behave more confidently in group interactions and exhibit empathetic and cooperative attitudes (Susanto, 2024). Although the study in question does not directly address the concept of social self-efficacy, the development of social skills and communication is considered a component of an individual’s perception of adequacy in social settings (Bandura, 1997; cited in Schwarzer, 2014). Therefore, it is predicted that the empowering effect of physical activity on social skills will indirectly increase perceptions of social self-efficacy.

*H_8_*: Quality of life (job satisfaction) mediates the relationship between physical activity and social self-efficacy. Numerous studies emphasize that physical activity increases individuals’ quality of life and job satisfaction, which in turn strengthens their sense of confidence and competence in social relationships. Job satisfaction is considered a psychosocial mechanism that can shape social behaviors and perceptions of self-efficacy through the meaning, commitment, and psychological balance individuals experience in their work life (Judge et al., 2001). In particular, the mediating role of job satisfaction has often been used to explain the effect of physical and psychological variables on social outcomes. For example, Warr (2011) stated that job satisfaction, one of the components of quality of life, can play a balancing role between an individual’s level of social participation and their perception of self-efficacy. Therefore, this study predicts that quality of life (the job satisfaction subscale) functions as a mediating variable that strengthens the effect of physical activity on social self-efficacy.

The aim of this study is to reveal the interaction process between physical activity level and quality of life (job satisfaction) in the context of empathic and social self-efficacy models among teachers and to test the mediating role of job satisfaction in this process. This approach aims to contribute both theoretically and practically to the literature on psychosocial resources and teacher well-being.

## 2 Materials and methods

### 2.1 Objectives and significance of the research

The aim of this study is to examine the mediating role of quality of life in the relationship between teachers’ participation in physical activity and empathic self-efficacy and social self-efficacy. The study investigated whether participation in physical activity has direct and/or indirect effects on self-efficacy, and whether quality of life serves as a mediating variable in this relationship.

### 2.2 Population and sample

A power analysis was conducted using the G*Power 3.1 program to determine the adequacy of the sample size for the study. In the mediation model based on multiple regression analysis (Model 4), a medium effect size (f^2^ = 0.15), a 95% confidence level (*α* = 0.05), and 80% test power (1-β = 0.80) were assumed for the two predictor variables (physical activity and quality of life). The analysis determined that meaningful results could be obtained with a minimum of 107 participants. The sample size of 688 participants in the study was well above this requirement, and the statistical power of the analyses was high (1**-**β > 0.99).

The research group consisted of a total of 688 teachers actively working in the Eastern Anatolia region, selected through simple random sampling and voluntarily participating in the study. The participants’ ages ranged from 23 to 40, with a mean age of 29.3 ± 7.79. Simple random sampling is one of the most basic probability sampling methods, in which each individual in the population has an equal chance of being selected (Büyüköztürk et al., 2017; Karasar, 2018). The participants’ personal information is presented in Table 1.

**Table 1:** Information regarding participants’ individual characteristics.

Variables	Groups	Frequency (n)	Percentage (%)	X̄
Gender	Male	372	54.1	
	Female	316	45.9	
Age				29.3
Marital status	Married	256	37.2	
	Single	432	62.8	
Field	Physical education	155	22.5	
	Other	533	77.5	
Years of experience	0–5 years	358	52.0	
	6–10 years	175	25.4	
	11–15 years	82	11.9	
	16 years and above	73	10.6	
Educational level taught	Elementary school	173	25.1	
	Middle school	216	31.4	
	High school	299	43.5	
Having children	Yes	227	33.0	
	No	461	67.0	
Physical activity level (based on MET calculation)	Low	285	41.4	
	Moderate	301	43.8	
	High	102	14.8	

### 2.3 Data collection tools

For data collection in this study, the personal information form prepared by the researchers, the International Physical Activity Questionnaire (IPAQ), the Quality of Life Scale for Employees (ProQOL-R-IV), and the Perceived Empathic and Social Self-Efficacy Scale were used.

### 2.4 Personal information form

Data regarding participants’ demographic characteristics, including gender, age, and marital status, were collected using a personal information form prepared by the researchers.

#### 2.4.1 International Physical Activity Questionnaire (IPAQ)

The International Physical Activity Questionnaire**-**Short Form (IPAQ**-**Short Form), adapted into Turkish by Öztürk (2005), was used to determine individuals’ physical activity levels. The questionnaire consists of seven items that assess individuals’ walking, moderate and vigorous physical activities, and sitting times over the past 7 days. The data obtained were calculated in metabolic equivalent minutes per week (MET**-**min/week), with higher scores indicating higher levels of physical activity. In the validity and reliability analyses conducted by Ozturk (2005), the test–retest reliability of the short form was found to be r = 0.69, and the criterion validity was r = 0.30. These findings show that the Turkish version of the questionnaire is a valid and reliable data collection tool for determining physical activity levels.

#### 2.4.2 Quality of Life Scale for Employees (ProQOL-R-IV)

The Professional Quality of Life Scale (ProQOL**-**R**-**IV), developed by Stamm (2005) and adapted into Turkish by Yeşil et al. (2010), was used to determine participants’ quality of life levels. The scale consists of 30 items and three subscales: Compassion Satisfaction, Burnout, and Compassion Fatigue. Participants responded to each item on a six-point Likert scale ranging from “Never (0)” to “Very often (5).” However, in this study, only the Professional Satisfaction subscale was included in the analyses in accordance with the study hypotheses. This subscale measures individuals’ satisfaction with their profession and their positive feelings toward their work. In the Turkish adaptation study, the overall Cronbach’s Alpha coefficient for the scale was reported as 0.85, and 0.82 for the Job Satisfaction subscale.

#### 2.4.3 Perceived Empathic and Social Self-Efficacy Scale

The Perceived Empathic and Social Self-Efficacy Scale, developed by Di Giunta et al. (2010) and adapted into Turkish by Akın and Başören (2015), was used to measure individuals’ levels of empathic and social self-efficacy. The scale consists of 11 items and two subscales: Empathic Self-Efficacy and Social Self-Efficacy. Participants responded to each item on a five-point Likert-type scale ranging from “1=Not at all applicable” to “5 = Completely applicable.” Cronbach’s Alpha internal consistency coefficients were reported as 0.78 for the empathic self-efficacy subscale and 0.80 for the social self-efficacy subscale. In this study, considering the structural characteristics of both subscales, the Empathic and Social Self-Efficacy variables were analyzed in separate models.

### 2.5 Research model

This research is descriptive in nature and was conducted within the framework of a correlational survey model. The correlational survey approach aims to determine the relationships between variables or the degree to which variables change together (Karasar, 2018). The study examined the mediating role of quality of life in the effects of physical activity level on empathic and social self-efficacy.

To properly test the mediating effects, the total score for physical activity (MET total) was not used directly in a categorical form (“low,” “medium,” “high”) in the analyses. This is because mediation analyses (Hayes, 2018) are based on linear relationships between continuous variables, and converting the total score into categorical form could distort the variance structure of the model. Therefore, the MET total score (continuous variable), representing each individual’s physical activity level, was directly included in the analysis.

In addition, in this study, the quality of life variable was measured based on the occupational satisfaction subscale determined in line with the research hypotheses, rather than the entire scale. Although the Quality of Life Scale consists of 30 items, 10 items representing the professional satisfaction subscale were included in the mediation analysis. Thus, the effects of quality of life on empathic and social self-efficacy were examined in the context of individuals’ perceptions of job and professional satisfaction.

**Figure 1:**
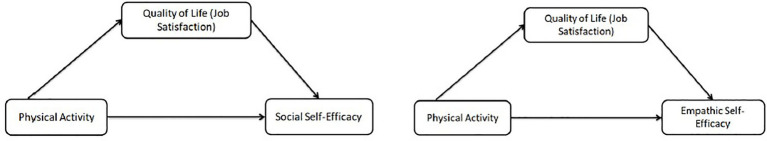
Research model.

Two separate mediation models were created accordingly. In the first model, empathic self-efficacy was considered the dependent variable, while in the second model, social self-efficacy was considered the dependent variable. In both models, quality of life was used as a mediating variable through the occupational satisfaction subscale. Hence, the indirect effects of quality of life on each type of self-efficacy were tested separately (see Figure 1).

### 2.6 Analysis of data

Data collected from teachers were transferred to the SPSS software, and descriptive analyses were performed. After validating the scale structures, skewness and kurtosis values were examined for the normality assumption, and these values were found to be between **−**3 and +3 (Jondeau and Rockinger, 2003). Pearson correlation analysis was conducted among the variables, and the coefficients were interpreted as follows: 0.00**–**0.10 = insignificant, 0.10**–**0.39 = weak, 0.40**–**0.69 = moderate, 0.70**–**0.89 = strong, and 0.90**–**1.00 = very strong (Schober et al., 2018).

Confirmatory Factor Analysis (CFA) was applied to test the construct validity of the scales used in the study (Şimşek, 2007). CFA analyses were performed using the AMOS 24.0 software package. Subsequently, the PROCESS Macro (Model 4) developed by Hayes (2018) was used to test the mediating effects predicted in the research hypotheses. The significance of the tested effects at a 95% confidence interval was evaluated based on whether zero was included within the lower and upper bounds and whether the *p*-value was less than 0.05 (Arbuckle, 2011).

## 3 Result

Participants consisted of 54.1% males (*n* = 372) and 45.9% females (*n* = 316), with a mean age of 29.3 years. In terms of marital status, 62.8% of the teachers were single (*n* = 432), while 37.2% were married (*n* = 256). Regarding field of specialization, 22.5% of the participants (*n* = 155) were working in physical education, whereas 77.5% (*n* = 533) were employed in other subject areas. When years of professional experience were examined, more than half of the teachers had 0–5 years of experience (52.0%, *n* = 358), followed by 6–10 years (25.4%, *n* = 175), 11–15 years (11.9%, n = 82), and 16 years and above (10.6%, n = 73). With respect to the level of education taught, 43.5% of the teachers were working at high schools (*n* = 299), 31.4% at middle schools (*n* = 216), and 25.1% at elementary schools (*n* = 173). Additionally, 33.0% of the participants (*n* = 227) reported having children, while 67.0% (*n* = 461) indicated that they did not have children. Based on MET calculations, physical activity levels were classified as low for 41.4% (*n* = 285), moderate for 43.8% (*n* = 301), and high for 14.8% (*n* = 102) of the teachers.

As part of the reliability analyses of the scales, the AVE (Average Variance Extracted) and CR (Composite Reliability) values were calculated for each factor. The online AVE and CR calculation system developed by Aydoğdu (2023) was used for these calculations. An AVE value greater than 0.50 was considered an acceptable threshold for convergent validity, while a CR value greater than 0.70 was considered an acceptable threshold for composite reliability (Fornell and Larcker, 1981). The skewness and kurtosis values of the variables ranged between −3 and +3, indicating that the normal distribution assumption was met (Jondeau and Rockinger, 2003). The Cronbach’s alpha coefficient ranges from 0 to 1, and a value of at least 0.70 is considered acceptable for reliability (Field, 2009; Hair et al., 2019). McDonald’s omega (*ω*) is another reliability coefficient calculated in addition to Cronbach’s alpha. This coefficient also takes values between 0 and 1, and the closer it is to 1, the more reliable the scores obtained (Lucke, 2005) (Table 2).

**Table 2:** Interrelationships between variables, reliability coefficients (α, ω, CR), average variance extracted (AVE), and skewness-kurtosis values.

	Variables	(α)	(ω)	CR	AVE	Skewness/kurtosis	M	SD	1	2
1	Empathic self-efficacy	0.87	0.87	0.87	0.53	−0.095/2.40	4.06	0.63	-	
2	Social self-efficacy	0.84	0.85	0.84	0.52	−0.097/1.99	4.10	0.67	0.70*	-
3	Job satisfaction	0.92	0.93	0.92	0.56	−0.051/−0.287	3.81	0.79	0.53*	0.59*

1 = Empathic Self-Efficacy; 2 = Social Self-Efficacy; 3 = Job Satisfaction. *p < .01 (two-tailed).

According to the PROCESS Model 4 mediation analysis, the total effect of physical activity (total MET) on empathic self-efficacy was significant (*β* = 0.0001, *p* = 0.005). When quality of life (job satisfaction) was included as a mediating variable, this direct effect lost its significance (*β* = 0.0001, *p* = 0.084). The effect of physical activity on quality of life (*β* = 0.0003, *p* = 0.014) and the effect of quality of life on empathic self-efficacy (*β* = 0.211, *p* < 0.001) were both significant. Since the indirect effect obtained through the bootstrap method (*β* = 0.0001, BootLLCI = 0.0000, BootULCI = 0.0002) did not fall within the zero range, it was concluded that quality of life (job satisfaction) plays a significant mediating role in the relationship between physical activity and empathic self-efficacy (Hayes, 2018) (Table 3).

**Table 3:** Mediating effect analysis (empathic self-efficacy).

Path	B	SE	t	*p*	LLCI	ULCI	R^2^
a (Physical activity → job satisfaction)	0.000	0.000	2.457	**0.0142**	0.0001	0.0005	M: R^2^ = 0.0087
b (Job satisfaction → Empathic self-efficacy, controlling for X)	0.211	0.012	16.448	< 0.001	0.1861	0.2366	Y: R^2^ = 0.2911
c’ (Physical activity → empathic self-efficacy, controlling for M)	0.0001	0.000	1.732	0.0836	0.000	0.0001	**-**
Indirect effect (a × b)	0.0001	Boot SE = 0.000	**-**	Bootstrap significant	0.000	0.0002	**-**
Total effect (c)	0.0001	0.000	2.782	**0.0055**	0.0000	0.0002	Y: R^2^ = 0.0112

Bold values indicate statistically significant effects at *p* < .05.

The PROCESS Model 4 mediation analysis showed that the total effect of physical activity (total MET) on social self-efficacy was significant (*β* = 0.0001, *p* = 0.019). When quality of life (job satisfaction) was added as a mediating variable, this direct effect lost its significance (*β* = 0.0000, *p* = 0.270). The effect of physical activity on quality of life (*β* = 0.0003, p = 0.014) and the effect of quality of life on social self-efficacy (*β* = 0.209, *p* < 0.001) were significant. Since the indirect effect obtained using the bootstrap method (*β* = 0.0001, BootLLCI = 0.0000, BootULCI = 0.0002) did not fall within the zero range, it was concluded that quality of life (job satisfaction) serves as a significant mediating variable in the relationship between physical activity and social self-efficacy (Hayes, 2018) (Table 4).

**Table 4:** Mediating effect analysis (social self-efficacy).

Path	B	SE	t	*p*	LLCI	ULCI	R^2^
a (Physical activity → job satisfaction)	0.000	0.000	2.457	0.0142	0.0001	0.0005	M: R^2^ = 0.0087
b (Job satisfaction → social self-efficacy, controlling for X)	0.208	0.010	19.122	< 0.001	0.1871	0.2300	Y: R^2^ = 0.3532
c’ (Physical activity → social self-efficacy, controlling for M)	0.000	0.000	1.103	0.2702	0.000	0.0001	**–**
Indirect effect (a × b)	0.000	Boot SE = 0.000	**–**	Bootstrap significant	0.000	0.0002	**–**
Total effect (c)	0.000	0.000	2.345	0.0193	0.000	0.0002	Y: R^2^ = 0.0080

### 3.1 The first model (empathic self-efficacy)

*H_1_*: Physical activity level has a significant and positive effect on quality of life (job satisfaction).

*H_2_*: Quality of life (job satisfaction) has a significant and positive effect on empathic self-efficacy.

*H_3_*: Physical activity level has a significant and positive effect on empathic self-efficacy.

*H_4_*: Quality of life (job satisfaction) mediates the relationship between physical activity and empathic self-efficacy.

### 3.2 The second model (social self-efficacy)

*H_5_*: Physical activity level has a significant and positive effect on quality of life (job satisfaction).

*H_6_*: Quality of life (job satisfaction) has a significant and positive effect on social self-efficacy.

*H_7_*: Physical activity level has a significant and positive effect on social self-efficacy.

*H_8_*: Quality of life (job satisfaction) mediates the relationship between physical activity and social self-efficacy.

**Figure 2:**
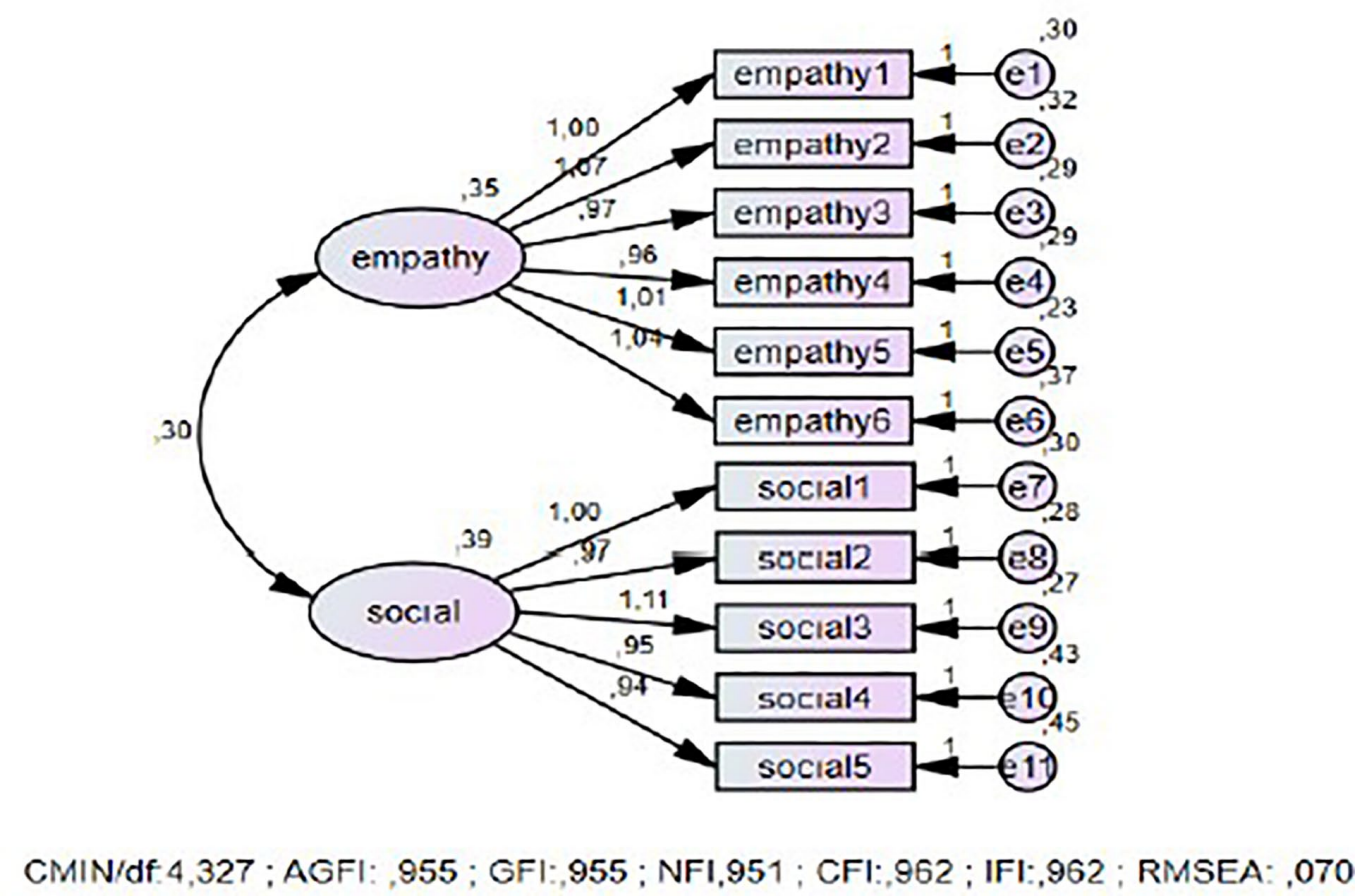
First-level CFA for the perceived empathetic self-efficacy and social self-efficacy scale.

It is recommended that the CMIN/df ratio be less than 5 (Kline, 2015). For RMSEA, values of 0.08 and below indicate a good fit, while values between 0.08 and 0.10 indicate a poor fit (Hooper et al., 2008). For the GFI, CFI, NFI, and IFI indices, a value of 0.90 indicates an acceptable fit, a value of 0.95 indicates an excellent fit, and for AGFI, a value of 0.85 indicates an acceptable fit (Bentler and Bonett, 1980; Hu and Bentler, 1999; Marsh et al., 2006; Schermelleh-Engel et al., 2003) (Figure 2).

**Figure 3:**
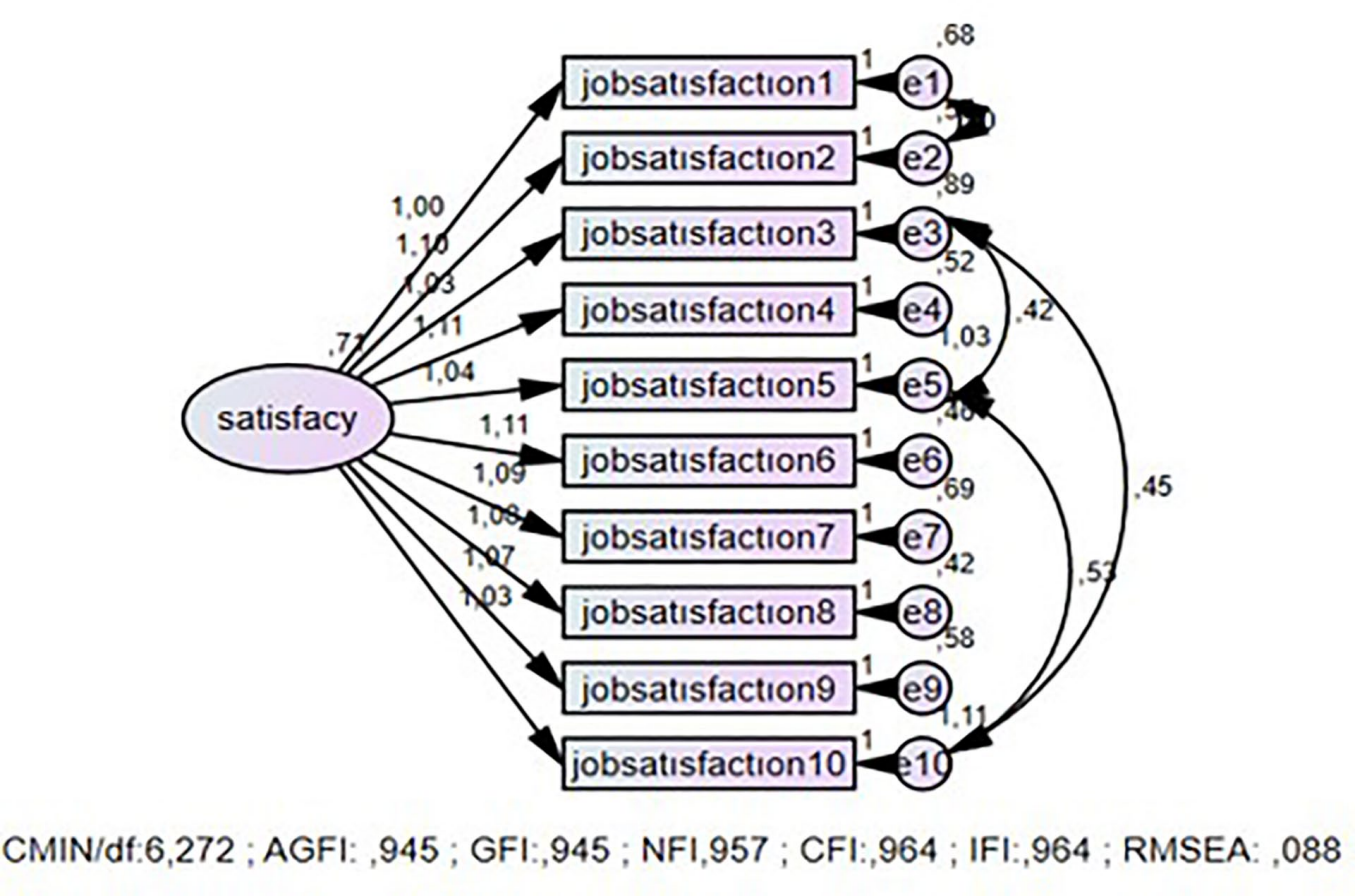
First-order CFA for the job satisfaction subfactor of the quality of life scale for employees.

In line with Kline (2016) and Schermelleh-Engel et al. (2003), it has been stated that high χ^2^/df and RMSEA values may lead to the rejection of the model, especially in large samples. Therefore, a general evaluation should be made by considering other fit indices (CFI, IFI, GFI, NFI, etc.). Consequently, the fact that the incremental fit indices are at an acceptable level in the current study indicates that the model generally demonstrates a good fit (Figure 3).

## 4 Discussion

This study aimed to examine the mediating role of quality of life in the professional satisfaction subscale in the relationship between teachers’ physical activity levels and their perceptions of empathic and social self-efficacy. Analyses conducted using PROCESS Model 4 revealed that physical activity had significant total effects on both types of self-efficacy; however, when quality of life was added to the model as a mediating variable, the direct effects lost their significance. These findings suggest that quality of life (job satisfaction) may play a full or partial mediating role in the context of empathic and social self-efficacy.

For example, a study examining the effect of physical activity on mental health outcomes through self-efficacy emphasized that self-efficacy plays a central role in such mediating processes (White et al., 2024). Furthermore, mediating effects have been observed in studies revealing that self-efficacy levels are shaped by variables such as physical activity, self-perception, or psychological resilience (Peng et al., 2025). In this context, the findings are consistent with similar models in the literature and offer a unique contribution in the context of teachers.

Below, comments are provided for each hypothesis, comparisons are made with similar studies in the literature, and explanatory conclusions are presented.

*H_1_*: Physical activity level has a significant and positive effect on quality of life (job satisfaction).

This hypothesis (H_1_) was supported: physical activity level was found to have a positive and significant effect on job satisfaction (*β* = 0.0003; *p* = 0.014). This finding supports the view that high levels of physical activity can foster positive psychological awareness, energy, and mood among individuals. In this regard, physical activity can be a valuable resource for improving teachers’ perceptions of satisfaction in the work environment. Although a study involving teachers reported positive relationships between physical activity and psychological well-being, the reliability of the study by Guo and Jiang (2023) is questionable. Additionally, there are studies focusing on the mediating role of variables such as self-perception and psychological well-being in the effect of physical activity on self-efficacy (Guo et al., 2024). For example, in the study by White et al. (2024), the mediating role of variables such as self-efficacy, self-esteem, and self-perception in the relationship between physical activity and mental health outcomes was strongly supported.

*H_2_/H_6_*: Quality of life (job satisfaction) has a significant and positive effect on empathic and social self-efficacy.

Quality of life (job satisfaction) was found to have a significant and positive effect on both empathic (*β* = 0.211, *p* < 0.001) and social self-efficacy (*β* = 0209, *p* < 0.001), and our hypotheses were accepted. These findings indicate that individuals with high levels of satisfaction tend to perceive themselves as more competent in social and emotional interactions. Moreover, considering the positive influence of teachers on society, it is reasonable to infer that teachers with a high quality of life may also develop empathy skills and contribute to raising psychologically healthy individuals. The literature includes studies suggesting that teachers’ professional satisfaction levels are linked to their ability to establish empathetic relationships with students (Skaalvik and Skaalvik, 2017). Furthermore, a study examining the relationship between school culture and teacher empathy demonstrated that the empathy variable played a significant role in the model where school culture affected job participation (Fu et al., 2022). These results can also be interpreted through the lens of social cognitive theory: highly satisfied teachers may possess a stronger capacity for social interaction by maintaining emotional balance, which in turn may enhance their perceptions of social self-efficacy.

*H_3_/H_7_*: Physical activity level has a significant and positive effect on empathic and social self-efficacy.

In the first stage, the total effects of physical activity on empathy (*p* = 0.005) and social self-efficacy (*p* = 0.019) were found to be significant. This finding partially supports our hypothesis that physical activity may directly affect psychological resources. However, when quality of life was added to the model as a mediating variable, the significance of the direct effects decreased or disappeared. This indicates that the effects were largely mediated by quality of life (Jiang and Zhang, 2025).

Such patterns of strong mediation are frequently observed in psychological process models. For example, studies have argued that the effects of physical activity on self-efficacy occur through variables such as motivation, well-being, or self-perception (Peng et al., 2025; Ke et al., 2024). A similar process was identified in the present study.

*H_4_/H_8_*: Quality of life (job satisfaction) mediates the relationship between physical activity and empathic self-efficacy, and between physical activity and social self-efficacy.

In both models, quality of life (job satisfaction) had significant indirect effects on the relationship between physical activity and self-efficacy (empathic/social), as the confidence intervals in the Bootstrap analysis did not include zero. Notably, when the mediating variable was added to the model, the direct effects lost their significance. This finding indicates a full or near-full mediation effect, consistent with existing literature on mediation models, which often report that psychosocial effects occur indirectly rather than directly (Hayes, 2018). In other words, for physical activity to enhance perceptions of self-efficacy, teachers may need to experience a high level of life satisfaction. This result reveals that job satisfaction acts as a psychological bridge. Similarly, although studies focusing on psychological variables mediating the relationship between teachers’ physical activity and self-efficacy are limited, findings suggest that variables such as well-being, self-esteem, and motivation play mediating roles in this process. Indeed, in health psychology studies, self-efficacy and self-perception have frequently been evaluated as mediating mechanisms. For example, Zhang et al. (2025) demonstrated that self-efficacy played a significant mediating role in the relationship between physical activity and mental health. Similarly, Gao et al. (2024) proposed a model explaining the effect of physical activity on teachers’ health perceptions through body image and self-efficacy.

## 5 Conclusion

This study contributes meaningfully to the literature by examining the interaction between physical activity, job satisfaction, and perceptions of self-efficacy in the context of teachers through a mediation model. Specifically, it demonstrates that the mediating role of quality of life functions as an important psychological bridge even when direct effects are not apparent. Within education systems, the role of teachers extends beyond imparting knowledge and is also considered highly significant for students’ emotional and social development.

However, certain considerations should be noted. The β values obtained are expressed as very small numbers, such as “0.0001.” In this regard, the magnitude of the effects should be interpreted with greater sensitivity, and the practical significance of the findings should remain open to discussion. Furthermore, the participant group is limited to teachers working in a specific geographical region; therefore, results obtained in different regions or cultural contexts may vary.

Schools and educational institutions should provide infrastructure and opportunities that encourage teachers to engage in physical activity. Initiatives such as sports facilities, walking groups, and fitness programs can enhance teachers’ professional satisfaction by supporting their physical and psychological resources. Such supportive environments may play an indirect yet effective role in strengthening teachers’ perceptions of self-efficacy.

Workplace improvement strategies aimed at increasing professional satisfaction levels can also be developed. Practices such as collecting teacher feedback, improving working conditions, and strengthening perceptions of fairness may reinforce teachers’ commitment to their institutions and enhance their psychological well-being.

Psychosocial support programs, along with empathy and social skills training, can positively influence teachers’ perceptions of self-efficacy by strengthening their emotional and social competence.

In future research, variables such as social support and leadership behaviors can be incorporated into the model to explore the mechanisms of influence in greater depth. Such analyses can increase the explanatory power of theoretical models by revealing how relationships among physical activity, quality of life, and self-efficacy vary according to contextual conditions.

### 5.1 Limitations of the study

The sample was limited to teachers from the Eastern Anatolia Region of Turkey; therefore, generalizations to other regions or cultures should be made with caution. Additionally, the cross-sectional design prevents drawing definitive conclusions about causal relationships.

## Data Availability

The raw data supporting the conclusions of this article will be made available by the authors, without undue reservation.
